# The Effect of Video-Based Preanaesthetic Preparation Versus Conventional Approach on Parental Anxiety in Paediatric Dental Procedures: A Prospective Cohort Study

**DOI:** 10.7759/cureus.26768

**Published:** 2022-07-12

**Authors:** Neeli S Phaneendra, Karan Singla, Indu M Sen, Shyam Meena, Manoj K Jaiswal

**Affiliations:** 1 Department of Anaesthesiology, Post Graduate Institute of Medical Education and Research, Chandigarh, IND; 2 Department of Oral and Maxillofacial Surgery, Post Graduate Institute of Medical Education and Research, Chandigarh, IND

**Keywords:** parents’ knowledge, parents, dental anxiety, video recording, paediatrics, operating room

## Abstract

Background

Parental anxiety has been identified as a risk factor affecting the behaviour of children before operative intervention. A preanaesthetic visit is a standard component of preoperative preparation, which may reduce parental anxiety. The use of audiovisual aids to demonstrate the conduct of anaesthesia may help improve parental education and reduce anxiety.

Patient and methods

We analysed data from a prospective randomised trial conducted at a tertiary care hospital. Parents of children posted for day care dental procedures were enrolled in the study. Children could be of either gender, aged 2-6 years, and categorised as American Society of Anesthesiologists Physical Status (ASA-PS) 1 or 2. Parents’ anxiety regarding the surgical and anaesthesia procedure was assessed using the Amsterdam Preoperative Anxiety and Information Scale (APAIS). The first APAIS scoring was recorded on arrival in the preoperative holding area. Thereafter, the participants were randomly allocated into two groups; one group was shown a short video on a smartphone of a dental operating theatre (OT), dental chair and anaesthesia equipment (SPG group), while the other group was verbally explained the dental procedure (conventional management or CM group). The second APAIS scoring was done in the postoperative recovery area one hour after the procedure. Demographic characteristics, socio-economic conditions and history were recorded. Anxiety scores were compared between the two groups, and any change was analysed.

Results

Seventy parents were included in the study, with 36 randomised to the SPG group and 34 to the CM group. Both groups were comparable in terms of demographic characteristics. There was a statistically significant decrease in anxiety scores in the SPG group, from a mean of 25.47 at the preoperative assessment to 14.92 at the postoperative timepoint (p<0.001). In the CM group, the mean APAIS score decreased from 25.26 to 24.56 (p=0.059).

Conclusion

There was a significant reduction in anxiety scores in the postoperative period among parents who were shown an operating room video in the preoperative period.

## Introduction

Dental anxiety (DA) remains a barrier to seeking oral health services in a proportion of the population [1]. Dental anxiety (DA) is defined as a state of apprehension that something dreadful is going to happen in relation to dental treatment, and it is coupled with a sense of losing control. About 6%-15% of the world’s population suffers from avoidance of dental care due to increased dental anxiety. Excessive parental anxiety has also been identified as a preoperative risk factor in children, and this can affect the behaviour of the child before operative intervention [2-5]. A preanaesthetic visit is a standard component of preoperative preparation and reduces the anxiety of parents of children posted for elective procedures. Providing appropriate information about anaesthesia, surgery and postoperative recovery will reduce anxiety in parents. With the use of mobile technology, it is feasible to prepare videos and share them with the parents at the site of care. The purpose of this study was to observe the effect of video-based parental education in reducing dental anxiety.

## Materials and methods

The study was conducted in the Oral Health Sciences Centre of Post Graduate Institute of Medical Education and Research, Chandigarh, India. Institutional ethics committee approval was obtained (INT/IEC/2019/002678: reference number NK/5717/MD/568), and the study was prospectively registered under the Clinical Trial Registry of India (CTRI/2020/04/024595). Parents of children aged 2-6 years of American Society of Anesthesiologists Physical Status (ASA-PS) 1-2 scheduled for any dental procedure on a day care basis were considered eligible to participate. Parents/guardians who refused to consent or were unable to understand the study protocol were not included. In the preoperative area, parents were instructed to fill a proforma containing questions about demographic characteristics, socio-economic conditions and relevant birth and medical history of their child. A thorough physical evaluation including airway assessment and systemic examination was conducted in the preoperative holding area, and findings were noted in the proforma (Annexure 1). The anxiety scoring of parents was done using the Amsterdam Preoperative Anxiety and Information Scale (APAIS) [6] by a blinded investigator. This is a reliable, validated assessment tool that consists of a 6-point questionnaire with five possible responses for each question: (1) not at all, (2) somewhat, (3) moderate, (4) moderately high and (5) extremely high (Annexure 2). Scores range from 6 to 30. Scores > 11 indicated high dental fear, and scores < 11 indicated low dental fear. Subsequently, using computer-generated block randomization, the participants were divided into two groups: the smartphone group (SPG group), in which the participants were shown a video of a dental operating theatre (OT) focusing on a dental chair, equipment, airway management devices, vaporizers, suction devices and IV fluid set, and the conventional management group (CM group), in which the participants were verbally explained about the dental procedure in the preoperative holding area.

Conduct of anaesthesia

All patients received an oral formulation of midazolam at a dose of 0.5 mg/kg as premedication. The children were taken inside the dental operating room by the attending anaesthesiologist, and standard ASA monitoring was initiated. Anaesthesia was induced with a fentanyl injection at 1 µg/kg; sevoflurane was titrated to effect. Intravenous atracurium 0.5 mg/kg was administered to facilitate endotracheal intubation, and isoflurane in an oxygen/N_2_O mixture was used for maintenance. Intraoperative vitals and the duration of anaesthesia and surgery were noted. All children received intravenous paracetamol (15 mg/kg) and ondansetron 0.1 mg/kg soon after the induction of anaesthesia.

Postoperative, vital parameters (HR, BP and SpO2), oral bleeding and the number of vomiting episodes were assessed at two-hourly intervals until the children were deemed fit to be discharged. The second APAIS scoring for the enrolled parents was done after one hour in the postoperative recovery area.

Statistical analysis

Data were analysed using SPSS version 22.0 (IBM Corp., Armonk, NY, USA) and Microsoft Excel 2010 (Microsoft Corp., Redmond, WA, USA). The normalcy of all continuous variables, such as age, SBP, DBP, SpO2, body mass index (BMI) and anxiety scale score, was checked using the Kolmogorov-Smirnov test. Data are expressed as mean ± standard deviation (SD) for normally distributed continuous variables or median (first quartile, third quartile) for non-normally distributed continuous variables. Frequencies (percentages) were used to calculate categorical variables such as gender, ASA grading and comorbidities.

The sample size calculation was based on the study conducted by Assuncão et al. [7]. To achieve a power of 80% and a level of significance of 0.05, 32 patients needed to be enrolled in each group. Assuming a dropout rate of 10%, the total number of patients enrolled was 70.

## Results

**Figure 1:**
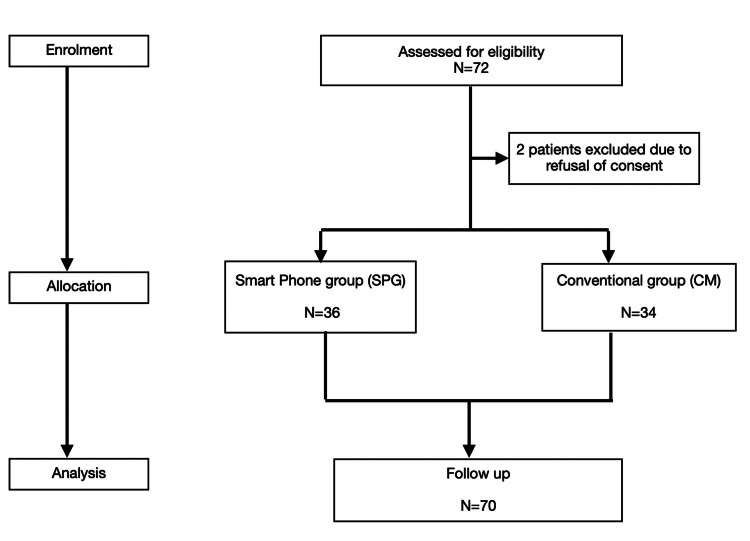
CONSORT flow diagram SPG: smartphone group, CM: conventional management

A total of 72 parents were assessed for eligibility. Two patients were excluded as they could not understand the questionnaire. Seventy parents were randomised to either the SPG group (n=36) or the CM group (n=34) (Figure 1).

 Parental age, educational status, occupation, socio-economic data and type of family structure are shown in Table 1.

**Table 1:** Demographic and educational status of parents SPG: smartphone group, CM: conventional management

Variables	SPG (n=36)	CM (n=34)
Father’s age (years) (median (range))	30.3 (26-38)	26.8 (35-35)
Mother’s age (years) (median (range))	26.02 (20-32)	22.1 (18-32)
Father’s education		
Illiterate	0 (0%)	2 (5.9%)
Primary school	0 (0%)	0 (0%)
Middle school	6 (16.7%)	8 (23.5%)
High school	3 (8.3%)	3 (8.8%)
Intermediate	0 (0%)	1 (2.9%)
Graduate	17 (47.2%)	13 (38.2%)
Postgraduate	10 (27.8%)	7 (20.6%)
Mother’s Education		
Illiterate	7 (19.4%)	13 (38.2%)
Primary school	3 (8.3%)	4 (11.8%)
Middle school	3 (8.3%)	2 (5.9%)
High school	1 (2.8%)	2 (5.9%)
Intermediate	0 (0%)	0 (0%)
Graduate	17 (47.2%)	11 (32.4%)
Postgraduate	5 (13.9%)	2 (5.9%)
Father’s occupation		
Bank clerk	3 (8.3%)	3 (8.8%)
Bank manager	4 (11.1%)	2 (5.8%)
Business	2 (5.5%)	7 (20.5%)
Cleaner	3 (8.3%)	6 (17.6%)
Clerk	1 (2.7%)	1 (2.9%)
Driver	8 (22.2%)	9 (26.4%)
Engineer	6 (16.6%)	2 (5.8%)
Receptionist	1 (2.7%)	0 (0%)
Teacher	7 (19.4)	3 (8.8%)
Technician	1 (2.7%)	1 (2.9%)
Mother’s occupation		
Bank employee	9 (25%)	4 (11.8%)
Engineer	1 (2.7%)	1 (2.9%)
Homemaker	7 (19.4%)	11 (32.4%)
Maid	11 (30.5%)	14 (41.1%)
Receptionist	3 (8.3%)	0 (0%)
Saloon	1 (2.7%)	0 (0%)
Teacher	4 (11.1%)	4 (11.8%)
Family status		
Rich	0 (0%)	0 (0%)
Upper middle class	14 (38.9%)	8 (23.5%)
Lower middle class	8 (22.2%)	8 (23.5%)
Poor	14 (38.9%)	18 (52.9%)
Below poverty line	0 (0%)	0 (0%)

Demographic data including age, gender, weight, height and the BMI of the children were comparable in both groups (Table 2).

**Table 2:** Demographic details of the children Values expressed as absolute numbers, percentages, mean, median and range SPG: smartphone group, CM: conventional management, BMI: body mass index

Variables	SPG (n=36)	CM (n=34)
Gender (male/female)	26 (72.2%)/10 (27.8%)	26 (76.6%) /8 (23.5%)
Age (years)	3.86+1.62	3.03+0.94
Weight (kg)	17 (9-20)	18 (10-21)
BMI (kg/m^2^)	16.4 (14.9-18.6)	15.8 (14.2-19.3)

Sixty-five children in this study belonged to ASA-PS 1, and five patients belong to ASA-PS 2. Five patients had associated comorbidities, autism in three patients and cerebral palsy (CP) in two participants.

The majority of the parents in both groups were graduates or postgraduates and lived in nuclear families. According to the modified Kuppuswamy classification [8], about 38.9% of the parents in the SPG group and 52.9% of the patients in the CM group belonged to the lower socio-economic class, while 61.1% in the SPG group and 47% in the CM group belonged to the middle class. Among the enrolled participants, five reported a history of previous anaesthesia exposure. Vomiting episodes in the post-anaesthesia care unit (PACU) requiring rescue anti-emetic were noted in 7.1% of the patients, although intravenous ondansetron was administered to all the patients. None of the patients required hospital admission.

APAIS score

**Figure 2:**
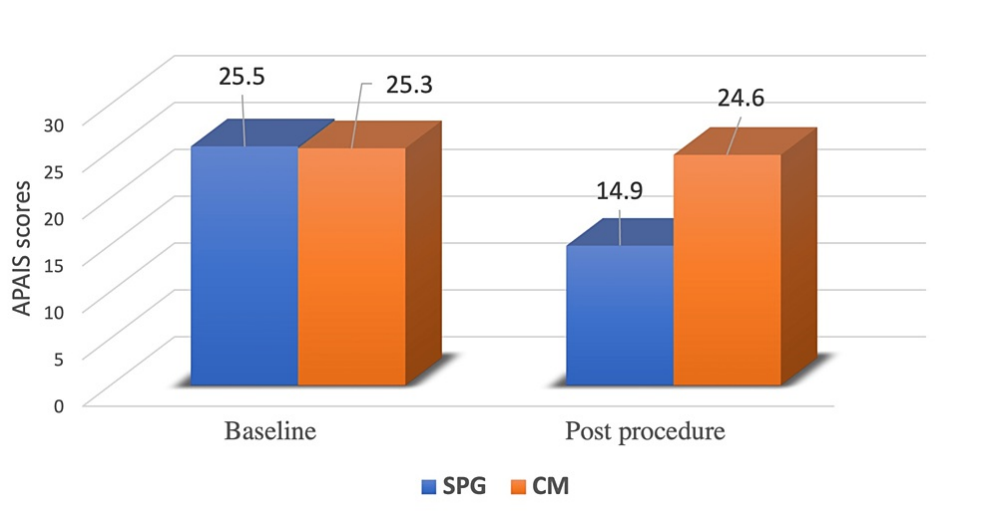
APAIS scores in the two groups APAIS: Amsterdam Preoperative Anxiety and Information Scale, SPG: smartphone group, CM: conventional management

In the SPG group, the mean APAIS total score decreased from 25.47 at the preoperative assessment to 14.92 at the postoperative timepoint (p<0.001). In the CM group, the mean APAIS score decreased from 25.26 at the preoperative examination to 24.56 at the postoperative evaluation (p=0.059) (Figure 2, Table 3).

**Table 3:** Comparison of the two groups in terms of change in the APAIS (total) over time (n=70) *Wilcoxon-Mann-Whitney U test APAIS: Amsterdam Preoperative Anxiety and Information Scale, SPG: smartphone group, CM: conventional management group, SD: standard deviation, IQR: interquartile range

APAIS (total)	Group				p-value*
	SPG		CM		
	Mean (SD)	Median (IQR)	Mean (SD)	Median (IQR)	
Preoperative	25.47 (2.96)	26.00 (2.00)	25.26 (3.00)	25.50 (4.50)	0.735
Postoperative	14.92 (2.84)	15.00 (2.25)	24.56 (3.45)	25.00 (5.50)	<0.001
Absolute change	10.56 (3.58)	11.00 (3.00)	0.71 (2.13)	0.00 (2.75)	<0.001
Percent change	40.7% (12.4)	42.6% (9.7)	2.7% (8.7)	0% (10.1)	<0.001
p-value for change in APAIS: total over time within each group (Wilcoxon test)	<0.001		0.059		
Overall p-value for the comparison of the change in APAIS: total over time between the two groups (generalized estimating equations method)	<0.001				

The parents of children with comorbidities did not demonstrate a higher anxiety score compared to those without any comorbidity (cerebral palsy (CP) patients: 26.5, no comorbidity: 25.5 and autistic children: 22) (Table 4).

**Table 4:** Comparison of the subgroups of variable comorbidity in terms of APAIS (total) (preoperative) (n=70) *Kruskal-Wallis test APAIS: Amsterdam Preoperative Anxiety and Information Scale, CP: cerebral palsy, SD: standard deviation, IQR: interquartile range, 𝛘^2^: chi-square

APAIS (total) (preoperative)	Comorbidity				
	Nil	Autism	CP	χ2	p-value*
Mean (SD)	25.49 (2.86)	22.00 (4.58)	26.50 (0.71)	2.214	0.331
Median (IQR)	26 (24-27)	21 (19.5-24)	26.5 (26.25-26.75)		
Range	14-29	18-27	26-27		

Parents belonging to lower socio-economic status demonstrated a higher mean APAIS score of 26.8 compared to 24.15 in the middle class (Table 5).

**Table 5:** Comparison of the subgroups on the basis of family income in terms of APAIS (total) (preoperative) (n=70) *Kruskal-Wallis test APAIS: Amsterdam Preoperative Anxiety and Information Scale, SD: standard deviation, IQR: interquartile range, 𝛘2: chi-square

APAIS (total) (preoperative)	Family income				
	Poor	Lower middle	Upper middle	χ2	p-value*
Mean (SD)	26.75 (2.06)	23.69 (3.79)	24.59 (2.56)	17.057	<0.001
Median (IQR)	27 (26-28)	24.5 (22.5-26)	25 (23.25-26.75)		
Range	18-29	14-29	19-28		

The mean APAIS score with a history of previous anaesthesia was 20 and with no history of anaesthesia was 25.9 (p<0.001).

## Discussion

The present study prospectively compared the effect of smartphone video-based preparation with conventional preparation on parental anxiety in children who underwent elective dental surgical interventions. The Amsterdam Preoperative Anxiety and Information Scale (APAIS) was used for assessment. The key finding was a significant reduction in anxiety score in the SPG group (mean preoperative APAIS score reduced from 25.47 to 14.96 one hour after the procedure) (p<0.001).

Cassady et al. studied the use of preanaesthetic video demonstration in parental education and anxiolysis [9]. Eighty-five parents of children undergoing day care otolaryngologic/ocular procedures, inguinal hernia repair or circumcision were randomised to attend video sessions. The experimental videotape group showed a significant reduction in anxiety scores compared to the control group (APAIS score from 22 to 17). The authors commented that parents’ knowledge about anaesthesia significantly improved in the intervention group, although these sessions were conducted 5-7 days prior to surgery. Whether these effects can be better sustained if sessions are conducted close to the perioperative period needs further evaluation. In the present study, all children underwent the same kind of procedure, i.e., dental interventions, and parents were assigned to the two groups on the day of surgery after the completion of baseline APAIS assessment proforma. All the participants interacted with the same investigator who was not aware of the baseline and post-procedure parental APAIS scores until the completion of the study. A significant reduction in scores compared to the conventional preparation group indicates that the parents benefitted when the video was shown on the day of the procedure. Moreover, since the video was prepared using a smartphone device by the investigators themselves, no professional cost was involved.

McEwen et al. enrolled 111 parents of children up to 16 years posted for elective day care surgeries under general anaesthesia [10]. Fifty-five parents were shown a short information video about perioperative procedures, while another 56 parents served as controls. All participants completed the APAIS questionnaire on the day of surgery, just before entering the operating theatre (OT) and after the procedure. The difference between pre-intervention (14.47) and post-intervention (12.37) scores was significant compared to the control group (13.68 to 13.25).

A systematic review by Kim et al. on the effect of technology-based preoperative preparatory interventions on perioperative parental anxiety concluded that video preparation programmes provided reasonable information for the management of parental anxiety [11].

Another video intervention-based trial by Aranha et al. enrolling mothers of children from lower educational status found reduced anxiety scores using the State-Trait Anxiety Inventory (STAI) at the time of shifting to OT and six, 24 and 48 hours after surgery [12]. The study showed that there was a significant decrease in anxiety levels from admission to shifting to OT.

The limitation of the present trial is that long-term follow-up to determine postoperative maladaptive behaviour was not done. Another limitation is that although anxiety was measured using a validated APAIS questionnaire, quantification by measuring stress biomarkers (cortisol level estimation) was not done due to financial constraints. Further trials can be conducted with long-term follow-up and quantitative estimation of stress markers.

To conclude, the results of the present study demonstrate that viewing a preanaesthetic educational video about paediatric anaesthesia and dental operating room setup facilitates parental education and significantly reduces anxiety. Further studies can be conducted where the actual conduct of anaesthesia and dental procedure is shown to the patients either as live recorded video or in a simulated environment. Similarly, behavioural modulations can be done using role-play techniques.

## Conclusions

To conclude, the results of the above investigation demonstrate that viewing a preanaesthetic educational video about paediatric anaesthesia and dental operating room setup facilitates parental education and significantly reduces anxiety associated with operative procedures. Further studies can be conducted where the actual conduct of anaesthesia and dental procedure is shown to the patients either as live video or in a simulated environment.

## Figures and Tables

**Figure 3:**
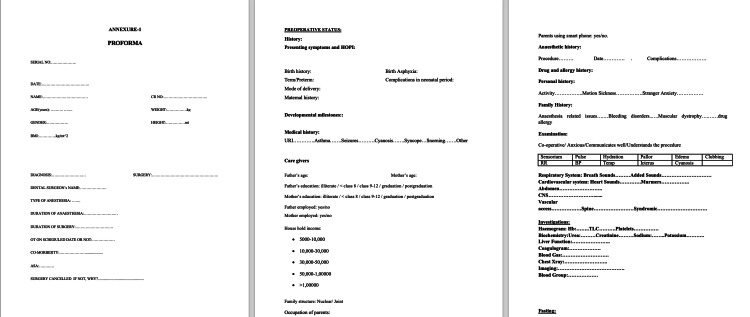
Proforma to be filled by the participants

**Figure 4:**
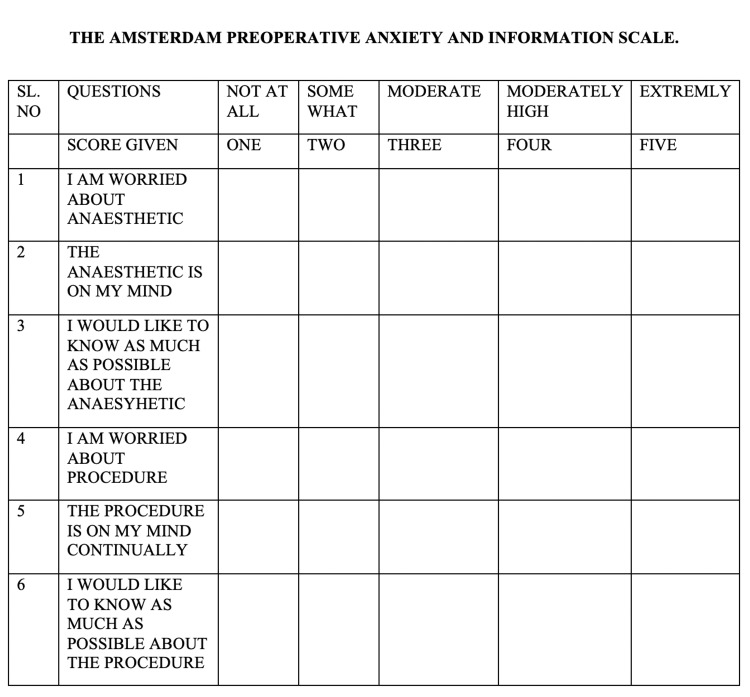
APAIS scoring system

## Appendices

Figure 3 shows the proforma containing questions on demographic characteristics, socio-economic conditions and relevant birth and medical history of the children.

Figure 4 shows the Amsterdam Preoperative Anxiety and Information Scale (APAIS) used in this study for the anxiety scoring of the parents.
